# Aortic arch pathologies – incidence and natural history

**DOI:** 10.1007/s00772-016-0155-5

**Published:** 2016-06-30

**Authors:** S. W. K. Cheng

**Affiliations:** Division of Vascular Surgery, Department of Surgery, The University of Hong Kong, Queen Mary Hospital, 102 Pokfulam Road, Hong Kong, China

**Keywords:** Aneurysm, Thoracic, Pathogenesis, Natural history, Etiology, Aneurysma, Thorax, Pathogenese, Natürlicher Krankheitsverlauf, Ätiologie

## Abstract

Endovascular interventions have made significant progress and are moving towards treating diseases of the aortic arch. Aortic arch pathologies incur substantial morbidity as well as short and long-term mortality but the progression is not well understood. This article reviews the current evidence on the natural history of aortic arch aneurysms and acute aortic syndromes, including penetrating ulcers, intramural hematomas, acute and chronic type B dissections. Risk factors for disease progression and mortality are also identified with special reference to vascular surgeons.

## Zusammenfassung

Bei den endovaskulären Interventionen hat es signifikante Fortschritte gegeben. Sie zielen auf die Behandlung von Erkrankungen des Aortenbogens. Pathologien am Aortenbogen haben sowohl kurzfristig als auch langfristig eine erhebliche Morbidität zur Folge, jedoch ist ihre Progression noch nicht ganz klar. Dieser Beitrag gibt einen Überblick über die aktuelle Evidenz bezüglich des natürlichen Krankheitsverlaufs von Aortenbogenaneurysmen und akuten Aortensyndromen, einschließlich penetrierende Ulzera, intramurale Hämatome sowie akute und chronische Typ-B-Dissektionen. Auch werden Risikofaktoren für die Krankheitsprogression und die Mortalität identifiziert, mit besonderem Augenmerk auf die Gefäßchirurgie.

## Introduction

Aortic arch pathologies are uncommon and often pose significant challenges to surgical or endovascular treatment. Many of the patients were either asymptomatic or otherwise had catastrophic life-threatening events. The true incidence and natural history of aortic arch diseases remain relatively unknown and treatment is often based on single center case series or large registries and there is reporting bias. Selection bias: patients selected for surgical treatment may be limited by anatomy and presentation. Poor outcome may not have been reported. Conditions affecting the aortic arch relevant to the vascular surgeon include degenerative aneurysms and the acute aortic syndromes (e.g. penetrating ulcers, intramural hematoma and aortic dissections).

## Aortic arch aneurysms

Degenerative aneurysms of the aortic arch are often asymptomatic. With the increase in usage of diagnostic imaging and aging populations, aortic arch aneurysms have been increasingly diagnosed. In a study of 590 consecutive patients undergoing surgery for thoracic aortic diseases, 78 % of whom had aneurysms, it was suggested that thoracic aortic pathology may be more common in patients with anatomical arch anomalies, in particular bovine arches and the patients are slightly younger (average age of 58.6 years for patients with aortic arch anomalies, compared to 62.4 years for those without arch anomalies) [1]. The increase in size of aortic arch aneurysms is an indolent process, with a reported mean annual growth rate of 0.07–0.2 cm/year [2]. Risk factors include age, female gender, chronic obstructive airway disease, hypertension, aortic diameter and a family history of aneurysms [3, 4]. There has been no prior study on the expansion rate or risk of rupture of true aneurysms specific to the aortic arch. The Yale University study [3] reported a growth rate of 1.2 mm per year in a mixed cohort with smaller aneurysms. A subsequent study from the same institution on 721 patients with thoracic pathology again reported an aortic expansion rate of 1 mm per year and a yearly rupture risk of 6.9 % in aneurysms exceeding 6 cm [5]. Prospective studies on descending thoracic and thoracoabdominal aneurysms reported a rupture risk of 23 % in 114 patients [4]. Similar earlier studies also reported a high incidence of rupture of up to 47 % in thoracic aneurysms treated medically and that the 5‑year survival was only 21 % in non-surgery groups and 50 % in all patients [6]. The yearly risk of aneurysm rupture increases generally with aneurysm size [5] and surgical intervention is therefore recommended for thoracic aneurysms that exceed a maximum diameter of 5.5–6.5 cm. The authors have recently conducted a study [7] on the natural history on 45 patients with isolated aortic arch aneurysms monitored with serial computed tomography (CT) scanning, excluding thoracoabdominal aneurysms and dissections. In a mean follow-up of 37 months the arch aneurysms were found to exhibit a tendency to linear growth with an average rate of 2.5 mm per year. Expansion rates were found to be associated with an aneurysm size >6.5 cm and also with hyperlipidemia [7]. While smaller aneurysms not included in this study may not be expanding at a similar rate, it is also possible that aortic arch aneurysm expansion is faster compared to rates reported for descending thoracic aneurysms. During surveillance in this study 22 % of aneurysms ruptured with an 80 % mortality. This rate of rupture is comparable to the 23 % rupture rate reported in previous natural history studies on descending and thoracoabdominal aneurysms [4]. On multivariate analysis of risk factors, only the aneurysm expansion rate of >5.5 mm per year was identified as an independent risk factor of rupture (67 % vs. 8 %, odds ratio of 1.43). A phase of rapid growth seems to occur before rupture and growth rate is the single most important predictor of rupture. In this series of patients more than one in five aneurysms ruptured in a mean follow-up period of 3 years, reinforcing the substantial risk of aneurysms in this location, where the arch is subjected to the highest blood pressure and hemodynamic stress. The maximum diameter of the aortic arch remains an important determinant of rupture.

## Acute aortic syndromes

Acute aortic syndromes comprise acute aortic dissections, intramural hematoma (IMH) and penetrating aortic ulcers (PAU). They can affect any part of the thoracic aorta, including the aortic arch. Penetrating ulcers and intramural hematomas are often considered variants of a true aortic dissection. It is estimated that one in eight patients with acute aortic dissection has an IMH or PAU.

## Penetrating ulcers

The PAUs are focal disruptions of the aortic intima breaching the internal elastic lamina to involve the media. They usually affect older patients, in atherosclerotic or diseased, dilated aortas and can be multiple. Other known risk factors for PAU include hypertension, smoking, coronary artery diseases, chronic obstructive airways disease and renal insufficiency. They can progress or be associated with intramural hematomas, rupture or develop into an acute aortic dissection. Stanson et al. reviewed 684 aortograms and found 16 patients with PAU in the descending thoracic aorta, giving an overall incidence of 2.3 % [8]. In a study using transesophageal echocardiograms in 194 patients for thoracic aortic disease, 12 (6.2 %) PAUs were found, 25 % of which occurred in the aortic arch [9]. Again the true incidence of PAU, particularly in the arch, remain unknown. With the increased use of imaging methods, occurrences as high as 11 % have been reported in patients presenting with acute aortic syndromes but other studies were not able to confirm this rising trend. A study on incidental findings of PAU in cardiac CT scans showed only 2 penetrating ulcers in 966 scans [10].

The natural history of PAU is also debated. The main complications are rupture, aortic dissection and aneurysm formation. It has been suggested that all PAU should be viewed as potentially dangerous and may lead to rupture [11]. If left untreated 30 % may enlarge to form a saccular aneurysm [12]. Supporters of this theory regarded PAU as progressive and any symptomatic ulcers should be treated by surgery or endovascular means as conservative therapy failed in up to 50 % of patients with PAU in the descending thoracic aorta [8, 13]. A PAU can be a precursor of aortic dissection. The Yale group found that 7.6 % of 198 patients with aortic dissections had a PAU and advocated aggressive repair in symptomatic patients. Of their patients 40 % of those treated with conservative means had aortic ruptures and needed emergency interventions [14]. Later the same investigators reasserted their notion by reporting an early rupture rate of 38 % and hospital mortality rate of 15 % in 26 patients with PAU [15]. Similarly, a Stanford University group of investigators treated 33 patients with PAU in 65 patients presenting with intramural hematoma whereby 48 % of those progressed and conservative treatment resulted in a 10 % mortality [16]. These reports suggested that those PAU associated with acute dissections or intramural hematoma tend to give a poorer prognosis. In symptomatic patients it is estimated that 25 % may progress to pseudoaneurysm formation and up to one  third result in ruptures [17]; however, others have shown that the natural history of PAU is more benign and a more conservative management may be adopted especially in asymptomatic patients. In a radiology study using CT scans on 33 ulcers, 64 % of lesions remained stable over a mean follow-up of 18 months. Out of an initial 56 lesions, 66 % were clinically stable. The authors concluded that most PAUs are asymptomatic and do not enlarge, while for the one third that progress the result is mild interval aortic enlargement (less than 1.5 times normal aorta diameter) [18]. No CT features were identified to predict progression. Cho et al. reviewed 105 PAU in the descending thoracic aorta and aortic arch, 85 of which were associated with intramural hematoma and 75 % were symptomatic. About one third of patients were treated with surgery with a mortality of 21 %. A total of 76 (72 %) of patients were treated medically with 3 deaths within 30 days. Generally in the vascular surgery community medical treatment of dissection is understood to be conservative treatment with medications on blood pressure control. Rupture at presentation predicted failure of medical therapy. They concluded that even in the acute setting many PAU can be managed non-operatively [19].

## Intramural hematoma

Caused by rupture of the vasa vasorum with bleeding into the aortic media, intramural hematoma (IMH) can be found in about 5–20 % of patients with acute aortic syndrome. This is a disease of the elderly and typically seen in patients over 70 years old. Risk factors include age, hypertension, atherosclerosis and IMH is believed to be a result of differential rigidity of medial and shear stress. The prevalence of IMH was reported to be 3.5 % from the International Registry of Acute Aortic Dissection (IRAD) registry [20]. There were discrepancies in other countries and the incidence of IMH has been reported to be higher in Asia: 44 % in Japan [21] and 28 % in Korea [22]. As a rule approximately one third of IMH will remain, one third will resolve and one third will progress to full aortic dissection.

If the IMH involves the aortic arch and ascending aorta the complication and mortality rates are high with medical management approaching that of a type A aortic dissection, whereas type B IMH seem to be more benign and most can be treated medically. The IRAD registry reported 107 patients with type B IMH where 88 % were treated medically with a hospital mortality of 7 %. During follow-up the mortality was 7 % and no adverse events were observed [23]. Symptomatic IMH has a higher incidence of rupture at presentation, up to 38 % [17]. In a meta-analysis of 143 reported cases IMH was associated with a mortality of 21 % [24]. Although there was no difference in the overall mortality between Stanford type A and B IMH, patients with type A IMH fared worse on medical treatment compared to surgery (36 % vs 14 % mortality) and also compared to type B IMH treated medically (14 %). In type B IMH medical and surgical outcomes were similar. The IRAD data also confirmed a higher mortality if the IMH involved the ascending aorta (39 %) compared with the descending aorta (8.3 %) [25]. Mortality is also higher if IMH is associated with penetrating ulcers. The natural history of IMH and rates of progression are not well known due to the difficulty in diagnosis and follow-up bias. An IMH may rupture, stabilize, regress or progress to dissection or aneurysm formation. Nienaber et al. reported a rate of progression of IMH to overt dissection, rupture or tamponade in 8 out of 25 patients (32 %) within 24–72 h, indicating the need for urgent intervention [26]. The progression of type A IMH to dissection in the literature ranged from 15 % to 85 % [27].

A Japanese registry compared 30 patients with type A IMH and 101 patients with acute type A dissection where all patients with type A IMH were initially treated medically. Failure of medical treatment and progression into dissection occurred in 43 % of patients. The overall hospital mortality of IMH (7 %) was significantly lower than the dissection group (24 %) and the long-term survival rates of IMH were also significantly better at 5 years (90 % vs 62 %), indicating that type A IMH have a better long-term prognosis than type A dissections [28].

Several risk factors have been proposed to predict progression of type A IMH to dissection. A dilated aorta and an ascending aorta diameter >5 cm were shown to be significant predictors of progression [29]. Other factors included the presence of a PAU, pleural and pericardial effusion, aortic regurgitation, persistent pai, and mediastinal hematoma [27]. In a CT analysis of 29 patients with IMH, Choi et al. identified type A, the maximum thickness of the IMH and compression of the true lumen to be indicative of active bleeding and increased risk of progression [30].

## Aortic dissection

There are many reports on the incidence of acute aortic dissections but the true incidence again is unknown due to the possibility of atypical presentations, undiagnosed cases and sudden death. The IRAD reported that up to 30 % were type B (not involving the ascending aorta) [31]. Population studies indicated that aortic dissection occurs in about 2–4 per 100,000 persons per year [32, 33]. The prevalence of aortic dissection ranged from 0.2 % to 0.8 % in autopsy studies [34]. There is some evidence to suggest that the incidence of aortic dissection is on the increase, up to 14 per 100,000 persons per year [35]. The incidence of aortic dissection after major cardiac surgery was estimated at 0.03–0.1 %, with aortic valve replacement carrying the highest risk.

In the northern Italy Emilia-Romagna Regional Database of hospital admissions, between 2000 and 2008 there were 1500 patients with acute aortic dissections, of whom 41 % were type A, 58 % type B and 10 % were complicated. They also reported an overall incidence of 4.7/100,000, with a male preponderance of 6.7 % vs 2.9 % females [34]. Notably almost 20 % of patients were 80 years of age or older. The overall in-hospital mortality was 27.7 %, 21.1 %, 26.9 % and 33 %, respectively for surgically treated type A, thoracic endovascular aortic repair (TEVAR) treated complicated type B and medically treated patients. Overall survival was at 62.2 %, 58.7 % and 55.8 % at 1, 2 and 3 years, respectively. Interestingly the authors found the annual incidence increased significantly with age. On the other hand, a Sino-RAD registry established in China in 2011 reported 1003 patients with acute dissections [36]. The patients in this registry were significantly younger (mean age of 51.8 years), consisting of more men (78 %), with a lower history of hypertension (59 %). Despite some criticism of selection bias and inadequate follow-up this showed that the true incidence of aortic dissection varies between countries and is subject to influence from socioeconomic status, primary healthcare and referral systems. The Interdisciplinary Expert Consensus Document on type B dissection pooled data from 63 studies and 6711 patients and reported an early mortality of 6.4 % with medical treatment and increased to 10.2 % with TEVAR and 17.5 % with open surgery for acute dissections. In chronic dissections of more than 6 weeks, a 5‑year survival of 60–80 % was reported. Recurrence of symptoms, aneurysms (>55 mm) or increase of aortic size >4 mm are predictors of poor outcome [37].

The fate of acute aortic dissections is better understood due to longer term follow-up studies. Type B dissections are considered mostly benign but death can occur in acute stages from rupture and malperfusion. The Yale experience confirms that uncomplicated type B dissections responded well to medical treatment with an early survival of 91 % and 66 % of patients had an uncomplicated follow-up [16]. The IRAD data from 1996 to 2013 on 1034 patients with type B dissection suggested an overall mortality of 10.6 % and identified variables adversely affecting hospital mortality, such as age, hypotension, periaortic hematoma, descending diameter ≥5.5 cm, mesenteric ischemia, acute renal failure and limb ischemia to be significant predictors of hospital death [38]. Refractory pain is also a predictor of in-hospital mortality an increase the risk from 1.5 % to 35.6 % [39], as well as patients older than 70 years [40]. Recently the IRAD investigators put forward a hyperacute phase from onset of dissection to 24 h in addition to acute (2–7 days), subacute (8–30 days) and chronic phases. They observed that the cumulative survival continued to significantly decline in all groups even up to 30 days, regardless of treatment modality (94–99 %, 82–93 %, 77–92 %, 73–91 %, respectively) [41]. Open repair of complicated acute type B dissection is associated with significant morbidity and mortality. A recent meta-analysis or contemporary series reported a pooled 30-day mortality of 19 % and a total neurological complication rate of 9.8 % [42]. While medical management of uncomplicated type B dissections can achieve a hospital mortality of 2.5–10 % [37, 42–44], on lon- term follow-up the false lumen may continue to enlarge resulting in aneurysm formation and is detrimental to survival. In 2006 IRAD reported a 3-year mortality of type B dissection of almost 25 %. Complicated type B dissection defined as malperfusion, hypertension despite medical therapy and increase in perioarotic hematoma and hemorrhagic pleural effusions were predictors of high hospital mortality [37] as well as 3‑year mortality [45]. The long-term survival of surgical and medically treated type B dissection patients are similar, with 5‑year survival of 44–83 % and 62–100 % respectively, with 5‑year freedom from aortic event rates of 59–68 % and 45–77 % respectively [37]. In a recent study Durham et al. described 198 patients with medically managed acute type B dissections and reported 58 % failed medical management at a mean follow-up of 4.2 years, including 12 % early failures within 2 weeks. Freedom from intervention was 77 % at 3 years and 74 % at 6 years, with an intervention-free survival of only 55 % at 3 years and 41 % at 6 years. Almost 30 % of patients subsequently underwent aorta-related interventions and 38 % died. They concluded that in the majority of patients with type B dissections medicinal therapy will fail over time [44].

Progressive dilatation and subsequent rupture of the false lumen are the common cause of long-term mortality. Risk factors for aortic diameter growth included male gender, an initial aortic diameter >40 mm, a large false lumen and a high blood pressure or pulse pressure. There is increasing evidence that a persistently patent or incompletely thrombosed false lumen is associated with a poorer prognosis [46, 47] but it is still uncertain whether a partially thrombosed false lumen carries a greater risk of progression than a completely patent false lumen.

## Conclusion

Aortic arch aneurysms, penetrating ulcers, intramural hematoma and dissections are increasingly diagnosed with better imaging procedures. The complete incidence and natural history is still relatively unknown but there is increasing evidence to make it possible to identify patients at risk of disease progression and ultimately mortality. With the increase in endovascular therapy, this information is invaluable in guiding surgeons to the best indications and timing for treatment.
